# Serum Has Higher Proportion of Janus Kinase 2 V617F Mutation Compared to Paired EDTA-Whole Blood Sample: A Model for Somatic Mutation Quantification Using qPCR and the 2^-∆∆Cq^ Method

**DOI:** 10.3390/diagnostics10030153

**Published:** 2020-03-12

**Authors:** Gustavo Barcelos Barra, Ticiane Henriques Santa Rita, Ana Luisa Santa Cruz Almeida, Rafael Henriques Jácomo, Lídia Freire Abdalla Nery

**Affiliations:** 1 Sabin Medicina Diagnóstica, SAAN, quadra 3, lote 145/185, Brasilia 70632-300, Brazil; ticihenriques@gmail.com (T.H.S.R.); santacruz.analuisa@gmail.com (A.L.S.C.A.); rhjacomo@gmail.com (R.H.J.); lidia@sabin.com.br (L.F.A.N.); 2 Post-Graduation in Health Science, University of Brasilia, Brasilia 70910-900, Brazil

**Keywords:** JAK2V617F, 2^-∆∆Cq^ method, allele-specific qPCR, relative quantification, myeloproliferative neoplasms

## Abstract

Detection of the Janus Kinase-2 (JAK2) V617F mutation is a diagnostic criterion for myeloproliferative neoplasms, and high levels of mutant alleles are associated with worse outcomes. This mutation is usually tested on blood DNA by allele-specific qPCR (AS-qPCR) and measured using absolute quantification. However, some automated DNA extractions co-extracts of PCR inhibitors from blood and qPCR absolute quantification need increased efforts in order to maintain standard curves. JAK2 V617F can also be detected in serum using droplet digital PCR (ddPCR), a specimen with less inhibitors and favorable to automated extractions, but ddPCR instruments are not wide available as qPCR thermocyclers. Here, we evaluate whether JAK2 V617F could be accurately quantified by AS-qPCR using the 2^-∆∆Cq^ method on blood DNA and validate the assay using gold-standard molecular diagnostic protocols. Next, we apply the validated method to assess if the mutation could be reliably detected/quantified in serum. JAK2 V617F could be quantified by AS-qPCR using the 2^-∆∆Cq^ method—the assay was highly accurate (bias of 1.91%) compared to a commercial kit, highly precise (total CV% of 0.40%, 1.92%, 11.12% for samples with 93%, 54%, and 2.5% of mutant allele), highly sensitive (limit of detection of 0.15%), and demonstrated a linear detection response from 1.1% to 99.9%. Serum presented a higher mutant allele burden compared to the paired whole blood (mean of 4%), which allows for an increased JAK2 mutant detection rate and favors increased JAK2 V617F high-throughput analysis.

## 1. Introduction

Myeloproliferative neoplasms (MPN) comprise of polycythemia vera (PV), essential thrombocythemia (ET), primary myelofibrosis (PMF), as well as chronic eosinophilic leukemia, and not otherwise specified and unclassifiable MPN [1]. This group of disorders is characterized by the constitutive activation of signal-transduction pathways responsible for hematopoiesis, which derives from a single somatically mutated hematopoietic stem cell. Phenotypically, they are described by hyperplasia of differentiated myeloid cells of one or more myeloid lineages and, consequently, have similar clinical features [2,3].

In 2005, four different groups identified a somatic point mutation in pseudokinase domain of Janus kinase-2 (JAK2) gene (JAK homology-2, JH2), which would then initiate the understanding of the molecular pathogenesis of those disorders. The mutation occurred in the autoregulatory JH2 tyrosine kinase domain located upstream of JH1 tyrosine kinase domain, leading to the loss of its inhibitory effect and, consequently, constitutive activation of JAK2 and downstream signaling molecules [4,5,6,7]. It is a G to T transversion at nucleotide 1849 in exon 14 of JAK2 gene located on chromosome 9p24, which leads to a valine (V) to phenylalanine (F) substitution at codon 617. JAK2 V617F mutation was present in over 95% of patients with PV and in approximately half of patients with ET and PMF [8]; and moreover, in 2008, the Word Health Organization included its detection as diagnostic criteria for MPN [9]. The clinical significance of JAK2 V617F quantification relied on the fact that high levels of mutant alleles are associated with increased risk of cardiovascular events, thrombosis and, in PV, to myelofibrotic transformation [10].

The specimen usually chosen to test for JAK2 V617F is peripheral blood using EDTA as an anticoagulant, because of its high abundance of DNA derived from cells of myeloid origin and sampling convenience when compared to bone marrow [11]. In 2018, Nystrand C. F. and colleagues described that the JAK2 V617F mutation could be reliably detected in serum using ddPCR. The authors also found a higher mutant allele burden in serum compared to paired peripheral blood samples, which may allow for increased mutant detection rates for serum. Reliable results were observed when more than 5 ng of serum DNA input was used in the ddPCR [12]. 

As leukocyte genomic DNA is released into serum during the coagulation [13], and blood clots continuously liberate genomic DNA molecules to this specimen overtime [14], both processes can deliberately be used to increase genomic DNA concentration in serum, which would guarantee the minimal amount of DNA ensuring reliable results. Additionally, some automated DNA extractions designed for serum/plasma co-extracts of PCR inhibitors when whole blood is used (e.g., Nuclisens Easymag System, Biomérieux, Marcy-l’Étoile, France) and additional purification steps are required to achieve amplifiable DNA [15]. These additional steps diminish the high-throughput of JAK2 V617F analysis in a clinical laboratory setting. Thus, serum is an advantageous alternative biological material compared to whole blood for JAK2 V617F detection/quantification. 

However, ddPCR instruments are not widely available for clinical and research laboratories as qPCR instruments, especially in developing countries. As such, in order to be more clinical useful, helping a great number of patients, the method should be migrated to qPCR. Today, AS-qPCR is the most widely performed method for JAK2 V617F detection/quantification due to its high analytical sensitivity (less than 1% of mutant alleles burden) [11]. The assay is mainly performed using two independent reactions, one specific for JAK2 V617 and other specific for JAK2 F617, and absolute quantification, which requires standard curves with known concentrations of the wild type and mutant JAK2 V617F alleles [16,17]. The main disadvantage of qPCR absolute quantification is the increased effort to generate and maintain standard curves [18].

Relative quantification is another method of analyzing qPCR data where the target of interest amount is presented relative to an internal calibrator or reference gene. A widely used method to calculate relative quantification is the 2^-∆∆Cq^ method, also referred as comparative Cq method, which is mostly used for gene expression analysis. The method returns the fold-change of the qPCR target of interest relative to reference target in two different samples (Sample A versus Sample B), so standard curves are unnecessary [18]. 

The 2^-∆∆Cq^ method has some premises: (a) The amplification efficiency of the target of interest and reference target must be similar and close to 1 and (b) the reference target abundance should be stable in sample A and B [18]. Thus, applying the 2^-∆∆Cq^ method to determine the JAK2 V617F mutant allele burden or quantifying any other somatic mutation is advantageous because it is easy to use and returns quantitative data (fold change), also working as a good orthogonal method for quantitative next-generation sequencing results validation (as required by current NGS validation protocols [19]). Today, a clear description of the 2^-∆∆Cq^ method application for JAK2 V617F mutation quantification (and other somatic mutation) is absent in the literature, especially if considering the inclusion of the 2^-∆∆Cq^ premises validation and the determination of the assay diagnostic parameters as: Limit of detection (LOD), limit of quantification (LOQ), linearity, precision and trueness.

Thus, the aims of this study were to evaluate whether JAK2 V617F mutant allele quantification could be accurately retrieved by AS-qPCR using the comparative Cq method on DNA extracted from whole blood, and to validate the assay using gold-standard molecular diagnostic protocols to find its diagnostic performance. Then, we applied the 2^-∆∆Cq^ method to assess if the mutation could be reliably detected and quantified in serum by AS-qPCR. 

## 2. Materials and Methods 

### 2.1. Ethics 

The FEPECS/SES-DF research ethical committee approved this study (registry CAAE 49481315.4.0000.5553, approval date: 09 November 2015) and written informed consent was obtained from each participant. 

### 2.2. Volunteers and recruitment

The study enrolled 27 healthy individuals with no clinical history of hematological disorders and 114 volunteers with a known status of a JAK2 V617F mutation (45 positives and 69 negatives). The patients with a known status of a JAK2 V617F mutation were obtained from our clinical laboratory service, which performed the assay according to Larsen, T.S., et. al. [20]. All patients who were referred to the laboratory for a JAK2 V617F test were invited to the study at the beginning of recruitment. Those patients who consented were invited to return for an extra collection of paired samples of serum and EDTA-whole blood. After the negative samples reached a reasonable number only the positive ones were contacted, especially those who presented some specific mutant allele burden. 

### 2.3. Sample Collection and Storage

Two tubes were drawn from venous blood of each participant, one Vacuette EDTA K2 whole blood 4mL tube and one Vacuette Z Serum Clot Activator 4 mL tube (Greiner-bio-one, Kremsmunter, Austria). Serum and whole blood tubes were transported to the processing unit refrigerated. This transportation could take 4–24 h. Upon arrival, serum was incubated for 48–60 h at room temperature to increase its DNA amount and then separated from the blood clot (2200× *g* for 10 min). Separated serum was stored at −20 °C until further processing. Whole blood was also stored at −20 °C upon arrival at the processing unit.

### 2.4. DNA Extraction 

DNA extraction from whole blood (200 μL) and serum samples (500 μL) was performed using Nuclisens Easymag System (Biomérieux, Marcy-l’Étoile, France) according to generic protocol 2.0.1 with the addition of 140 μL of magnetic silica particle suspension diluted in 600 μL of lysis buffer and eluted in 55 μL. Blood was premixed with lysis buffer (2 mL) and diluted silica (740 μL) off-board in a 15 mL tube to avoid inhibitor co-extraction; this step was not necessary for serum. Some DNA samples were also extracted using MagNA Pure 96 Instrument (Roche Diagnostics Ltd., Pleasanton, CA, USA) with the MagNA Pure 96 DNA and Viral NA Small Volume Kit for whole blood and MagNA Pure 96 DNA and Viral NA Large Volume Kit for serum, both eluted in 50 μL and processed according to manufacturer’s protocol. All extracted DNA samples were stored at −20 °C until further processing. 

### 2.5. DNA Quantification

DNA was quantified by qPCR absolute quantification using 65 base pairs RNAse P gene sequence as target. Primers and probes were previously described [14]. The qPCR reaction consisted in 7.5 μL of Maxima Probe/ROX qPCR Master Mix (2×) (Thermo Scientific, Waltham, MA, USA), 1.5 μL 10× RNAse P Prime Time Assay (Integrated DNA technologies, Coralville, IA, USA), 5 μL of DNA, 1 μL of PCR grade water, and the following thermocycling conditions: Denaturation for 10 min at 95 °C; followed by 40 cycles of 15 s at 95 °C and 15 s at 60 °C. The instrument used was StepOne^TM^ Real-Time PCR System (Applied Biosystems, Foster City, CA, USA). Two points standard curves (10^6^ and 10^3^ copies/reaction) were set up using ssDNA corresponding to the RNAse P gene target sequence (AGATTTGGAC CTGCGAGCGGGTTCTGACCTGAAGGCTCTGCGCGGACTTGTGGAGACAGCCGCTC) (Integrated DNA Technologies, Coralville, IA, USA). The equation describing the RNAse P calibration curve was Y = −3.26X + 29.75 (efficiency = 102.4% and R^2^ = 0.998). The DNA amount was calculated by the following formula: Number of RNAse P ssDNA copies returned from the qPCR plus the weight of human haploid genome in nanograms (0.0033 ng) divided by 2 to correct for dsDNA, plus the reaction DNA input volume (5 μL). The quantification by qPCR was used to reliably quantify short fragments DNA in serum. All DNA samples were normalized to 2.5 ng/μL and 10 μL was used in each AS-qPCR.

### 2.6. Quantification of JAK2 V617F Somatic Mutation using the 2^-ΔΔCq^ Method

JAK2 V617F wild type and mutant alleles were amplified by two independent AS-qPCR multiplex reactions, one specific for the wild type allele, and the other specific for the mutant allele. The RNAse P gene was co-amplified in each reaction to control if the inputted DNA was suitable for PCR and as reference target for the comparative Cq method, if desirable. JAK2 V617F wild type and mutant primers were described by Larsen, T.S., et.al [20]: Common primer: 5′-CTTTCTTTGAAGCAGCAAGTATGA-3, JAK2 V617F wild type primer 5′-GTAGTTTTACTTACTCTCGTCTCCACAtAC-3′, JAK2 V617F mutant primer 5′- GTAGTTTTACTTACTCTTGTCTCCACAtAA-3′ and probe 6FAM-TGAGCAAGC/ZEN/TTTCTCACAAGCATTTGGTTT-3IABkFQ (all purchased from Integrated DNA technologies, Coralville, IA, USA, as PrimeTime assays). Lower case letters indicate the deliberate mismatch introduced to enhance allele discrimination. JAK2 V617F amplicon size is 100 base pairs. The RNAse P gene primers were the same as described above (DNA quantification section). All AS-qPCR reactions were performed in StepOne^TM^ Real-Time PCR System (Applied Biosystems, Foster City, CA, USA) and consisted of 15 μL of Maxima Probe/ROX qPCR Master Mix (2×) (Thermo Scientific, Waltham, MA, USA), 3 μL of 10× JAK-2 Prime Time Assay and 1.5 μL 10× RNAse P Prime Time Assay and 10 μL of DNA normalized to 2.5 ng/ μL in the following thermocycling conditions: Denaturation for 10 min at 95 °C; followed by 45 cycles of 15 s at 95 °C and 60 s at 60 °C. Non-template control (NTC), positive, and negative controls were performed in each reaction. Threshold was always set to 0.1 DeltaRn in all qPCR experiments.

JAK2 V617 reaction was considered sample A and JAK2 F617 reaction was considered the sample B in the legacy 2^-ΔΔCq^ equation. The percentage of JAK2 V617F mutant allele was calculated using the equation: JAK2 mut % = 100% ÷ ((2 ^-(Cq JAK2 wt) - (Cq Jak2 mut)^) + 1), or JAK2 mut % = 100% ÷ ((2 ^-(Cq JAK2 wt - Cq RNAseP wt) - (Cq Jak2 mut - Cq RNAseP mut)^) + 1), if one would like to include RNase P in the calculus to control for the small variation in DNA quantity between wild type and mutant reactions (arose from pipetting two independent reactions). Derivation of these formulas can be found in the Supplementary Material. To demonstrate that both formulas reach very similar results DNA with 97.6%, 74.8%, 67.3%, 52.5%, 40.4%, 34.9%, 21.9%, 10.4%, and 4.8% of JAK2 mutant allele were quantified using both formulas.

### 2.7. Establishment of Cq Cut-Off Value

Twenty-seven healthy volunteers with no clinical history of hematological disorders were tested using the proposed method and the unspecific amplification of JAK2 V617F wild-type allele by the JAK2 V617F mutant primer was inspected, because it was observed in the seminal publication of these primers [20], and if present, the percentage of allele mutant was calculated using equation 4 described in the Supplementary Material.

### 2.8. Amplification Efficiency

JAK2 V617F wild type and mutant alleles amplification efficiencies were investigated by testing 10-fold or 2-fold serially dilution (4–5 dilution points) of some positive samples for JAK2 mutant allele encompassing a range of proportion considered low, medium and high in the clinical setting (from 6% to 96%) followed by the evaluation of the standard curve parameters, especially its slope, from which the amplification efficiency was derived. Slopes were compared using linear regression tools available in Graphpad Prism software version 6.0 (Graphpad, Inc., San Diego, CA, USA).

### 2.9. Limit of Detection (LOD) and Limit of Quantification (LOQ)

LOD was determined applying the probit regression analysis (Minitab Version 19.2.0.0.) to the results returned by the proposed method when applied to constructed DNA samples representing a 2-fold decreasing serial dilution of the JAK2 V617F mutant allele starting at 1.16% (1.16%, 0.58%, 0.29%, 0.145%, 0.073%, 0.036%, 0.018%, and 0.009% of mutant alleles). Protocol inspired from CLSI EP17-A2 [21], however, using artificial DNA that could have a different amplification behavior in the PCR. Each concentration point was constructed by spiking the appropriated amount of Ipsogen JAK2 MutaQuant (Qiagen, Hilden, Germany) positive control (100% mutant of alleles) into the negative DNA sample (100% of wild type alleles). Each concentration was tested nine times. The assay limit of quantification (LOQ) was also retrieved from this experiment by comparing the observed versus the expected amount (mean and SD) of mutant alleles using one-sample *t* test [Graphpad Prism software version 6.0 (Graphpad, Inc.)].

### 2.10. Precision

Three selected DNA samples with 93%, 54%, and 2.5% of mutant allele burden were submitted to the 2^-ΔΔCq^ method in duplicate for 9 consecutive working days (*n* = 18, for each concentration), and the mean, total, and between-day and within-run coefficients of variation were calculated. Protocol adapted from CLSI EP05-A2 [22].

### 2.11. Trueness

The proposed comparative Cq method for JAK2 V617F quantitative results were compared to the results observed by the commercial kit Ipsogen JAK2 MutaQuant (Qiagen, Hilden, Germany) kit using StepOne^TM^ Real-Time PCR System (Applied Biosystems, Foster City, CA, USA), executed according to manufacturer’s protocol. This kit used AS-qPCR and absolute quantification for JAK-2 mutant allele quantification. Total, positive, and negative agreement (qualitative analysis) were calculated as percentage of concordant result using 2 × 2 table. Pearson correlation, linear regression, and Bland–Altman analysis were used to calculate the correlation coefficient (*r*), the coefficient of determination (R^2^), and the agreement (bias) between both methods, respectively. Protocol adapted from CLSI EP15-A2 [23]. Calculations were performed using Graphpad Prism Software v6.0 (Graphpad, Inc) and the commercial kit was considered the reference method. The assay linearity was retrieved from this experiment (from linear regression results).

### 2.12. JAK-2 V617F Mutation Quantification in Paired EDTA-Whole Blood and Serum Using the 2^-ΔΔCq^ Method

Ninety-five paired samples of EDTA-whole blood and serum with known status for JAK-2 V617F mutation were submitted to the proposed method. EDTA-whole blood and serum sample volumes and DNA extraction details were described above in the DNA extraction section. The same analyses described in the session trueness were performed with the data obtained in this experiment: Total, positive and negative agreement, Pearson correlation, linear regression, and Bland–Altman analysis.

## 3. Results

### 3.1. Validation of the JAK2 V617F Mutant Allele Relative Quantification Using the 2^-ΔΔCq^ Method

**Figure 1:**
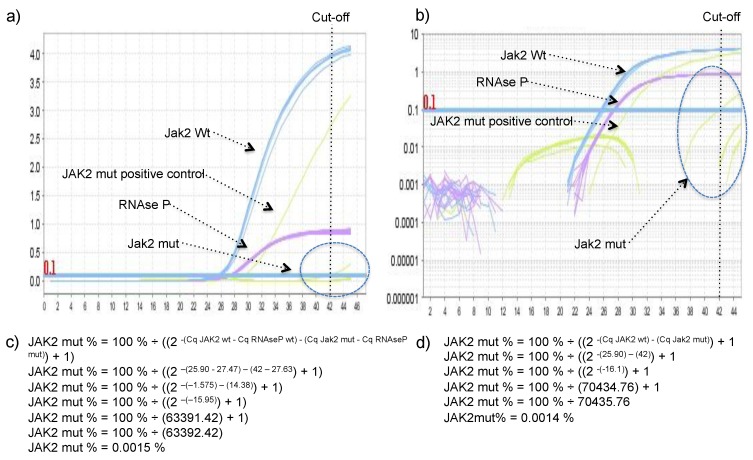
Depiction of Janus Kinase-2 (JAK2) wild type allele (blue), JAK2 mutant allele (yellow), and RNAse P (magenta) amplification curves in JAK2 V617F negative samples (*n* = 27) to illustrate the lack of amplification in most negative samples and the arbitrary established Cq cut-off value because one negative sample presented a Cq of 42: (**a**) Delta Rn in linear scale, (**b**) delta Rn in log scale. Examples of how raw data resulted in a particular mutant allele percentage: (**c**) Excepting RNAse P Cq, (**d**) including RNAse P in the calculation.

First, we determined if the proposed assay would return positive results for negative samples. Twenty-seven blood samples from healthy volunteers with no clinical history of hematological disorders were tested, 26 of them did not showed any positive signal after 45 PCR cycles, except one sample that presented a Cq of 42. This Cq value corresponded to 0.0015% of JAK2 mutant alleles when the proposed comparative Cq method was applied. A Cq cut-off value of > 42 was arbitrarily established, meaning that samples Cq higher than 42 were considered unspecific amplification and discarded. We conclude that the consistent unspecific amplification of JAK2 V617F wild-type allele by the JAK2 V617F mutant primer described in their seminal publication is not prevalent for the proposed method. Representative amplification plots and calculations can be found in Figure 1.

**Figure 2:**
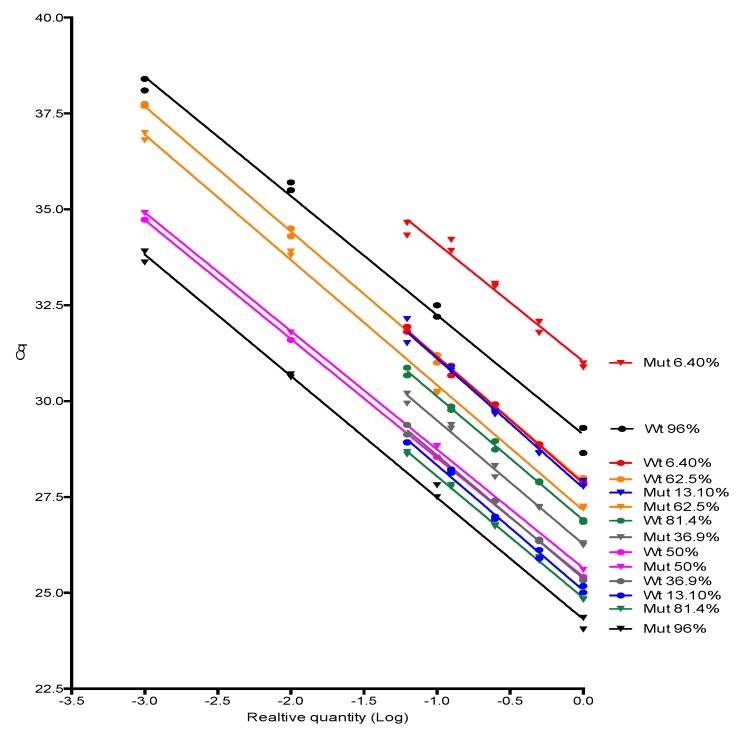
Linear regression depiction of JAK2 wild type allele (circles) and mutant allele (triangles) amplification when the proposed method was applied to 1:10 or 1:2 serial dilutions of DNA samples with distinct proportions of JAK2 mutant allele. We observed that both wt and mut standard curves were parallel (similar slopes) in all instances, satisfying the 2^-ΔΔCq^ premises. Each point represents a technical replicate.

Next, we investigated the JAK2 V617F wild type and mutant alleles amplification efficiencies applying a 1:10 or 1:2 serial dilution of DNA samples with distinct proportions of JAK2 mutant allele to the proposed method. The relative concentration of 1 was arbitrarily attributed to the first dilution point. The results can be found in Figure 2, which depicts standard curves and the associated linear regression of samples with distinct proportions of JAK2 mutant alleles, and in Table 1, which describes the corresponding AS-qPCR amplification efficiencies for wild type and mutant alleles. Wild type and mutant alleles AS-qPCR amplification efficiencies ranged from 100.5% to 109.7% and 97.9% to 111.7%, respectively. The intra-sample and inter-sample amplification efficiencies were not different in all instances (*p* values on Table 1), satisfying the 2^-ΔΔCq^ premises, so the method can be applied to the relative quantification of JAK2 V617F somatic mutation.

After that, we proved that both formulas JAK2 mut % = 100% ÷ ((2 ^-(Cq JAK2 wt) - (Cq Jak2 mut)^) + 1), or JAK2 mut % = 100% ÷ ((2 ^-(Cq JAK2 wt - Cq RNAseP wt) - (Cq Jak2 mut - Cq RNAseP mut)^) + 1) resulted in very similar outcomes. By submitting the Cq values of samples with 97.5%, 74.8%, 67.3%, 52.5%, 40.41%, 34.9%, 21.9%, 10.4%, and 4.8% of JAK2 mutant allele to both formulas, a Pearson correlation with *r* = 1, linear regression with R^2^ = 0.999 and Bland–Altman analysis with a bias of −0.61% (95% CI −2.5% to 1.3%) was observed, meaning a high agreement between them.

**Figure 3:**
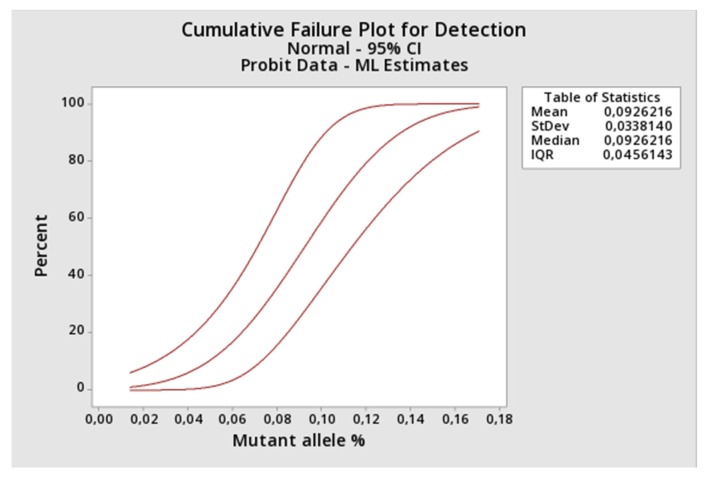
Probit regression analysis depicting the percentage of detection versus the JAK2 mutant allele (%). The LOD point is equal to the concentration at which 95% of the experiments gives a clearly positive signal.

Then, we determined the assay LOD, applying the proposed method to a 1:2 serial dilution of the Ipsogen JAK2 MutaQuant (Qiagen) positive control (100% mutant sample) spiked into the negative DNA sample. Appling the probit regression analysis to the results, a LOD of 0.15% (95%IC 0.12–0.21%) was returned, demonstrating that the assay has an excellent analytical sensitivity (Table 2 and Figure 3).

The limit of quantification could also be retrieved from this experiment by comparing the observed versus the expected result for each data point. Samples with 1.16%, 0.58%, 0.29%, 0.15% of mutant alleles (detected in at least eight of nine instances) returned the values of 1.03 ± 0.25% (*p* = 0.17), 0.19 ± 0.06% (*p* < 0.0001), 0.048 ± 0.037% (*p* < 0.0001), 0.0098% ± 0.0053 (*p* < 0.0001), indicating that the assay lost its linearity after 1.16%, so this value was considered the LOQ.

Next, samples with high (93%), medium (54%) and low (2.5%) JAK2 V617F mutant allele burden were tested in duplicate during nine consecutives working days. The observed mean, total CV%, between-day CV%, and within-run CV% indicated that the assay is highly precise along a wide detection range (variation lower that 12%) (Table 3).

**Figure 4:**
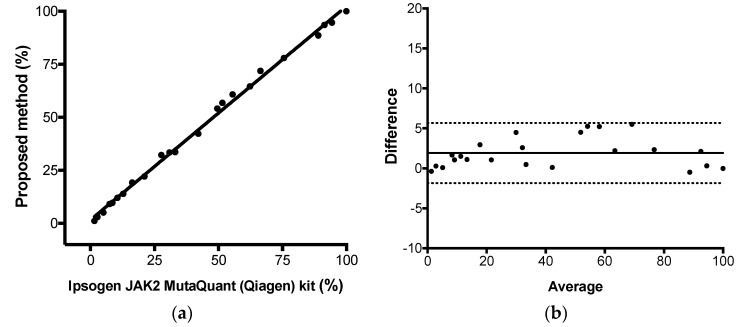
Proposed method trueness evaluation (2^-ΔΔCq^ method versus Ipsogen JAK2 MutaQuant (Qiagen) kit. (**a**) Linear regression (R^2^ = 0.996). (**b**) Bland–Altman analysis (bias of 1.91% (solid line) with 95% CI −1.87% to 5.6% (dashed lines)). Twenty-three selected positive samples encompassing the percentages range that could be observed in a clinical setting were tested (from 1.16% to 99.98%).

Finally, we evaluated the assay trueness by comparing the proposed method versus the Ipsogen JAK2 MutaQuant (Qiagen) kit head to head. The latter was considered the reference method. The test was performed using two reactions, one specific for the wild type and other for the mutant allele. However, the quantification was based on qPCR absolute quantification through standard curve. Twenty-three positive samples with JAK-2 V617F mutant alleles proportions ranging from 1.5% to 99.9% of mutant alleles and 19 negatives were submitted to both assays. We observed that the total, positive, and negative agreements were 100% between the two methods qualitatively (23 positives and 19 negatives results). Additionally, both methods presented similar quantitative results: Pearson correlation with *r* = 0.998 (*p* < 0.0001), linear regression with R^2^ = 0.996, and Bland–Altman analysis with a bias of 1.9% (95% CI −1.9% to 5.6%) (Figure 4a,b). These results indicate that the absolute quantification and the relative quantification of the JAK2 V617F were highly correlated and agree to each other very well (mean difference of 1.9%). The proposed method linearity could also be retrieved from this assay, as it returned a linear response from all tested range of mutant alleles (from 1.1% to 99.9%).

All the above-described experiments were executed on DNA extracted from whole blood.

### 3.2. Quantification of the JAK2 Using 2^-ΔΔCq^ in Paired Serum and EDTA-Whole Blood Samples

**Figure 5:**
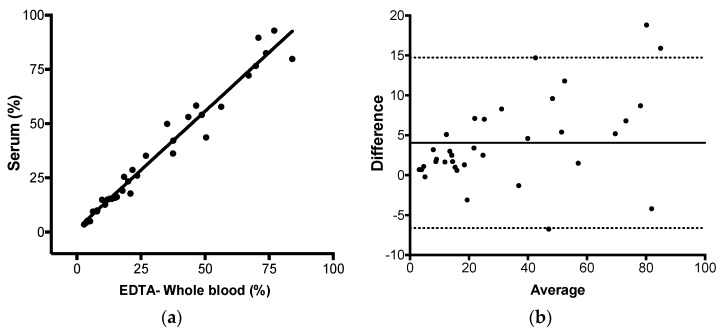
Comparison of JAK2 V617V mutant quantities in paired serum and EDTA-whole blood using 2^-ΔΔCq^ method. (**a**) Linear regression (R^2^ of 0.965). (**b**) Bland–Altman analysis (Bias of 4.058% (solid line) with 95% CI −6.6% to 14.7% (dashed lines)). Thirty-five positive samples with known results were included in this analysis.

After the assay validation, we evaluated if the JAK2 V617F mutation could be quantified in serum by AS-qPCR using the 2^-ΔΔCq^ method. EDTA-whole blood was the comparator and considered the reference specimen. Ninety-five selected samples with known JAK2 mutant allele burden were enrolled in this analysis. For DNA extraction, whole blood sample input was 200 μL and serum sample input was 500 μL. All DNA were normalized for 25 ng/reaction. The 2^-ΔΔCq^ method returned 35 positive results and 60 negative results for both specimens, so the qualitative total, positive, and negative agreement between serum and EDTA-whole blood were 100%. Pearson correlation, linear regression, and Bland–Altman analysis returned r of 0.982 (*p* < 0.0001), R^2^ of 0.965, and a mean difference (bias) of 4.0% (95% CI = −6.6% to 14.7%) (Figure 5a,b). Observing the data, it seems that the difference between serum and blood was higher in the samples with high percentages of mutant allele burden. As such, we performed subgroup analyses by dividing them into samples with less than 20% and more than 20% JAK2 mutant alleles in serum. Pearson correlation, linear regression, and Bland–Altman analysis returned r of 0.966 (*p* < 0.0001), R^2^ of 0.934, and a mean difference (bias) of 1.737% (95% CI = −0.83% to 4.30%) for the subgroup with less than 20% (*n* = 15) and r of 0.960 (*p* < 0.0001), R^2^ of 0.921, and a mean difference (bias) of 5.7% (95% CI = −7.2% to 18.8%) for the subgroup with higher than 20% of JAK2 mutant alleles (*n* = 20), demonstrating that samples with higher percentages of JAK2 mutant alleles contribute more extensively to the difference in the JAK2 mutant allele difference observed between the two specimens.

## 4. Discussion

In this study, we evaluated whether JAK2 V617F mutant allele quantification could be accurately performed by AS-qPCR using the comparative Cq method and defined the assay diagnostic performance using gold-standard molecular diagnostic validation protocols. Then, we tested if the method could reliably detect and quantify the mutation in serum.

First, we investigated whether the JAK2 V617F AS-qPCR generates a positive signal on the incorrect template (e.g., wild-type allele amplification by the mutant primer). The late amplification of the “wrong allele” by an allele specific primer is common in AS-qPCR [14], and it was observed in the seminal publication of these primers [20]. This outcome was generated because the mismatches that provide the allele discrimination do not impair, but delay the primer extension, allowing the genotyping [14]. In our hands, the delayed amplification of the wild-type allele by the mutant primer was rare, meaning that presence/absence of the target did not depend on a cut-off value, but on the presence or absence of amplification, which facilitates the achievement of a conclusion. However, because of that single false positive, a Cq cut-off value of 42 was established, and any Cq value higher than 42 was considered negative. The absence of the delayed amplification of the wild-type allele by the mutant primer could be secondary to the multiplex format of the assay, as RNAse P was co-amplified with JAK2 in all reaction tubes with Cq lower 30, which could somehow (e.g., production of pyrophosphate, FAM/HEX fluorescence crosstalk) further delay the unspecific amplifications, hence, it was not observed during the 45 cycles of the reaction, except in one sample.

Next, we investigate if the JAK2 V617F wild type and mutant allele have similar amplification efficiencies for the comparative Cq method to be applied. To our knowledge, this was the first time that the 2^-ΔΔCq^ method and AS-PCR were congregated to accurately retrieve the proportion of variant alleles in a sample. The JAK2 V617 reaction was considered as sample A and the JAK2 F617 reaction was considered as sample B in the legacy 2^-ΔΔCq^ equation. The unique difference between both reactions was the 3′-end nucleotide, hence, it was highly probable that both alleles showed similar amplification efficiencies. After testing several samples with distinct JAK2 mutant allele proportion, we empirically proved that JAK2 wild type and mutant primers had similar efficiencies, so the comparative Cq method premises was attended. Additionally, we demonstrated the relationship between the percentage of a somatic mutation and the Cq values of wild type and mutant alleles amplification by deriving the formula used in this study. The major advantage of this method was that it did not require standard curves. As DNA input of wild type and mutant AS-qPCR come from the same extraction tube, their Cq were the same or similar, and did not account for the JAK2 V617F mutant percentage. However, RNAse P Cq could control pipetting errors, so we considered it in all experiments of this study.

After that, we applied some gold-standard molecular diagnostic validation protocols to the assay to find its diagnostic performance. Parameters like trueness, total, between-days and within-run precisions, limit of detection, limit of quantification, and linearity were determined. The proposed assay demonstrated itself to be highly accurate compared to a commercial kit based on absolute quantification, highly precise along its linear range and highly sensitive, being capable to detect as little as 0.15% of mutant alleles in a sample. Correct quantitative results were found from 1.1%, result between 0.15% and 1.1% should be reported as detected, because the assay lost its linearity in this range. Taken together, the results showed that JAK2 V617F somatic mutations could be accurately quantified by AS-qPCR using the comparative Cq method.

Next, we evaluated if JAK2 V617F could be detected and reliably quantified in serum, as well as in whole blood, by AS-qPCR. Genomic DNA yield increases in serum incubated at room temperature, if the specimen remained in contact with the clot [14]. This strategy was used to ensure that serum always yielded the DNA amount necessary to the reaction (25 ng). However, serum DNA is highly fragmented, because blood DNAses are active in this specimen [24] and it’s probably of apoptotic origin [14]. As such, PCR products with small amplicon sizes amplify better than larger ones when applied to the DNA extracted from this specimen [14]. JAK2 V617F product used in this study had 100 base pairs, so amplicon size was not a concern in the detection/quantification of JAK2 alleles from serum DNA. Our results showed that not only could the JAK2 V617F mutation could be detected in serum by qPCR, but this specimen also presented a mean bias of 4.058% when compared to the paired peripheral blood sample. These results corroborate with the previous description using ddPCR [12], suggesting that the use of serum DNA may allow for increased mutant detection rates. Other serum advantages are: 1) It has less inhibitors than whole blood; 2) it can be directly used in qPCR [14]; and 3) it is more friendly most automated DNA extractions and additional purifications steps are not required to achieve amplifiable DNA [15], allowing increased high-throughput analysis. The downside of the incubation at room temperature to enrich genomic DNA in serum is the increase of the assay turn-around time. However, as soon as the sample could yield 25 ng of DNA, the assay can be performed. Sample input volume and DNA extraction elution volume can be adjusted to achieve this concentration, as soon as possible. Indeed, serum DNA amounts within 2h after phlebotomy ranges from 1500 to 7500 copies/mL or 5 to 25 ng/mL [13], so the necessary DNA yield can be achieved quickly. In this study, the incubation at room temperature was 48–60 h, after the sample arrival to the processing unit. Observing the data, it seemed that as allele burden increases, the difference becomes higher. Dividing allele burden into two categories, <20% (*n* = 15) and >20% (*n* = 20), there is a mean difference (bias) of 1.7% for the subgroup with less than 20% and 5.7% for the subgroup with higher than 20% of JAK2 mutant alleles. As such, samples with >20% contribute more extensively for the observed difference between serum and whole blood. Our hypothesis for the increased JAK2 V617F mutant allele burden in serum compared to the paired peripheral blood is that myeloid cells but not lymphoid cells, somehow, are preferentially disrupted during coagulation and during the ex-vivo incubation at room temperature, increasing the mutant allele proportion in this specimen.

Finally, the method and validation proposed in this study can be applied for other clinically relevant somatic mutations detected in the plasma of cancer patients, such as the gatekeeper mutation T790M in EGFR exon 20, the most common resistance mechanism to first- and second-generation tyrosine kinase inhibitors (erlotinib, gefitinib, and afatinib). Because of the availability of osimertinib, a drug that overcomes the T790M resistance mechanism, the detection of this gatekeeper mutation in plasma is of high importance in non–small-cell lung cancer [25,26]. Once 25 ng of DNA can be retrieved from the liquid biopsy samples, the assay can be performed, or smaller DNA inputs can be validated. Additionally, one application of the proposed AS-qPCR relative quantification using 2^-ΔΔCq^ is used as an orthogonal method for the validation of the NGS test for somatic mutations, as requested by current validations protocols [19], because the method demonstrates high sensitivity, reliability, is easy to implement, and the qPCR instruments are widely available.

## 5. Conclusions

Comparative Cq derived from allele-specific qPCR can be used for JAK2 V617F mutant allele quantification relative to wild type alleles. The assay is highly accurate (bias of 1.91%) compared to a commercial kit, highly precise (total CV% of 0.40%, 1.92=%, 11.1% for samples with 93%, 54%, and 2.5% of JAK2 mutant allele), and highly sensitive (LOD of 0.16%). The assay does not require standard curves and demonstrated a linear detection response ranging from 1.1% to 99.9%. When applied to serum, a higher mean allele burden of 4% compared to the paired whole blood was observed. Serum became an alternative specimen that could allow increased JAK2 mutant detection rates by qPCR and is friendly with the majority of most automated DNA extractions, favoring increased high-throughput analysis.

## Figures and Tables

**Table 1:** AS-qPCR amplification efficiencies of serial dilutions of DNA samples with distinct proportions of JAK2 mutant alleles.

Mutant Allele % (Dilution)	wt eff ^1^ (%)	Mut Eff (%)	*p* Value
96% (1:10)	109.7	106.8	0.61
81.4% (1:2)	104.1	106.9	0.45
62.5% (1:2)	102.4	102.4	0.95
50% (1:10)	109.5	110.02=	0.71
36.9% (1:2)	104.9	104.3	0.91
13.1% (1:2)	102.5	97.9	0.51
6.4% (1:2)	100.5	111.7	0.22

^1^ Eff—Amplification efficiencies, slopes of all linear regressions were not different (*p* = 0.18).

**Table 2:** Assay limit of detection (LOD) raw data and probability of detection (graphical results can be found in Figure 3).

Mutant Allele %	Tested (*n*)	Detected (*n*)	Detection (%)	Probability of Detection
1.160	9	9	100	1.000
0.580	9	9	100	1.000
0.290	9	9	100	1.000
0.145	9	8	88	0.939
0.073	9	4	44	0.280
0.036	9	0	0	0.047
0.018	9	0	0	0.013
0.009	9	0	0	0.006

**Table 3:** Assay precision evaluation.

Mutant Allele (%)	Mean (%)	Total CV (%)	Between-Day CV (%)	Within-Run CV (%)
93	93.5	0.40	0.2	0.3
54	54.1	1.9	1.1	1.5
2.5	2.76	11.1	9.2	6.2

## Supplementary Materials

Supplementary materials can be found at https://www.mdpi.com/2075-4418/10/3/153/s1.
