# Supplementary material for: Serum Has Higher Proportion of Janus Kinase 2 V617F Mutation Compared to Paired EDTA-Whole Blood Sample: A Model for Somatic Mutation Quantification Using qPCR and the 2-∆∆Cq Method
Source: Diagnostics (Basel). 2020 Mar 12;10(3):153. doi: 10.3390/diagnostics10030153 (PMC7151023; doi:10.3390/diagnostics10030153)
Supplement: Supplementary file 1 [file diagnostics-10-00153-s001.pdf]

Supplementary Materials:

The percentage of JAK2 V617F mutant allele was calculated using the equation: JAK2 mut % = 100% ÷ ((2 $^{-(\text{Cq JAK2 wt}) - (\text{Cq Jak2 mut})}$) + 1), or JAK2 mut % = 100% ÷ ((2 $^{-(\text{Cq JAK2 wt - Cq RNAseP wt}) - (\text{Cq Jak2 mut - Cq RNAseP mut})}$) + 1), if one would like to include RNase P in the calculus to control for small variation in DNA quantity between wild type and mutant reactions (arose from pipetting two independent reactions).

These formulas were derived from the following: JAK2 wild type and mutant allele relative quantification equals to percentage of JAK2 wild type allele divided by the percentage of JAK2 mutant allele (equation 1).

$$2^{-\Delta\Delta Cq} = \text{JAK2 wt \%} \div \text{JAK2 mut \%},$$

$$2^{-(\text{Cq JAK2 wt - Cq RNAseP wt}) - (\text{Cq Jak2 mut - Cq RNAseP mut})} = \text{JAK2 wt \%} \div \text{JAK2 mut \%}. \quad (1)$$

As DNA came from the same source (same normalized DNA), Cq RNAseP wt is equal to Cq RNAseP mut, so RNase P contribution to the calculus can be neglected because its does not account for the relative quantification and can be removed from the equation (equation 2). On the other hand, it can be maintained to control for small differences in the DNA amount used in each independent reaction.

$$2^{-(\text{Cq JAK2 wt}) - (\text{Cq Jak2 mut})} = \text{JAK2 wt \%} \div \text{JAK2 mut \%}. \quad (2)$$

The premise to apply the above-described comparative Cq method is that JAK2 wild type and mutant alleles could be considered the same PCR target, for so, they must show similar amplification efficiencies. Indeed, the unique difference between the two PCR reactions is the last 3'-prime nucleotide in each allele discrimination primer. Thus, a similar amplification behavior is expected for both reactions, however, it must be confirmed (see results). Additionally, percentage of JAK2 wild type allele plus the percentage of JAK2 mutant allele equals to 100% of JAK2 alleles in the sample (equation 3).

$$\text{JAK2 wt \%} + \text{JAK2 mut \%} = 100\%, \quad (3)$$

Substituting equation 3 on equation 2 results in the equation 4, which was used for JAK2 V617F mutant allele percentage calculation in this study:

$$2^{-(\text{Cq JAK2 wt}) - (\text{Cq Jak2 mut})} = (100\% - \text{JAK2 mut \%}) \div \text{JAK2 mut \%},$$

$$2^{-(\text{Cq JAK2 wt}) - (\text{Cq Jak2 mut})} = (100\% \div \text{JAK2 mut \%}) - (\text{JAK2 mut \%} \div \text{JAK2 mut \%}),$$

$$2^{-(\text{Cq JAK2 wt}) - (\text{Cq Jak2 mut})} = 100\% \div \text{JAK2 mut \%} - 1,$$

$$[(2^{-(\text{Cq JAK2 wt}) - (\text{Cq Jak2 mut})}) + 1] = 100\% \div \text{JAK2 mut \%},$$

$$\text{JAK2 mut \%} = 100\% \div [(2^{-(\text{Cq JAK2 wt}) - (\text{Cq Jak2 mut})}) + 1]. \quad (4)$$
